# Relation between anemia and blood levels of lead, copper, zinc and iron among children

**DOI:** 10.1186/1756-0500-3-133

**Published:** 2010-05-12

**Authors:** Amal A Hegazy, Manal M Zaher, Manal A Abd el-hafez, Amal A Morsy, Raya A Saleh

**Affiliations:** 1 Department of Community and Industrial Medicine, Faculty of Medicine, Alazhar University, Cairo, Egypt; 2 Department of Pediatric Medicine, Faculty of Medicine, Alazhar Univerisity, Cairo, Egypt; 3 Department of Clinical Pathology, Faculty of Medicine, Alazhar Univerisity, Cairo, Egypt

## Abstract

**Background:**

Anemia is a health problem among infants and children. It is often associated with a decrease in some trace elements (iron, zinc, copper) and an increase in heavy metals as lead. This study was done to determine the association of blood lead level > 10 μg/dl, with the increased risk to anemia, also, to investigate the relationship between anemia and changes in blood iron, zinc and copper levels, and measure lead level in drinking water.

The study is a cross-sectional performed on 60 children. Venous blood samples were taken from the studied population for estimating hematological parameters as well as iron and ferritin levels. The concentrations of zinc, copper, and lead were measured. The studied population was divided into anemic and non-anemic (control) groups. The anemic group was further classified into mild, moderate and severe anemia. The study subjects were also categorized into low and high blood lead level groups.

**Findings:**

Approximately 63.33% of children had blood lead levels ≥ 10 μg/dl. At the blood lead level range of 10-20 μg/dl, a significant association was found for mild and severe anemia. The blood level of iron and ferritin was found to be significantly lower in high blood lead level and anemic groups than those of the low blood lead level and control groups. Lead level in drinking water was higher than the permissible limit.

**Conclusion:**

Lead level ≥ 10 μg/dl was significantly associated with anemia, decreased iron absorption and hematological parameters affection. High blood lead levels were associated with low serum iron and ferritin. Lead level in drinking water was found to be higher than the permissible limits.

## Background

Deficiency of certain trace elements generally causes hypochromic microcytic anemia. Iron deficiency not only causes hypochromic microcytic anemia, but also increases the absorption of other elements such as lead (Pb) and cadmium (Cd). Therefore, in patients with hypochromic microcytic anemia, the serum levels of these elements may increase causing deterioration of anemia. Generally, heavy exposure to (Pb and Cd) causes hypochromic microcytic anemia [1]. Iron absorption occurs predominantly in the duodenum and jejunum. A number of dietary factors influence iron absorption, ascorbate and citrate increase iron uptake. Lead is a particularly pernicious element to iron metabolism, as it is taken up by the iron absorption machinery, and secondarily blocks iron through competitive inhibition. Furthermore, it interferes with a number of important iron dependent metabolic steps such as heme biosynthesis [2].

Lead poisoning has been a significant public health problem for centuries. In children, it is defined as a blood lead level equal to or greater than 10 μg/dl [3], it is also associated with adverse behavioral and developmental outcomes. However, a level < 10 μg/dl is considered unsafe [4].

Human exposure to lead occurs primarily through diet, air, drinking water and ingestion of paint chips where absorption increases mainly in persons suffering from iron and calcium deficiency [5].

Environmental lead exposure occurs from automobile exhaust in areas of the world where leaded gasoline is still used. At home, exposure among children may occur either due to ingestion of old leaded chips or pigments and glazes used in pottery [6].

For centuries, lead plumbing has helped in the contamination of drinking water and contributed to elevated blood lead concentrations in children [7].

The mobilization of heavy metals in the environment, due to industrial activities, is a serious concern due to their toxicity in humans and other forms of life [8]. These toxic metals (mercury, lead and cadmium) are called "the big three" due to their major impact on the environment. Where they tend to persist, circulating and eventually accumulate throughout the food chain [9].

Copper as an essential trace element exists in the diet, it is needed to absorb and utilize iron [10]. Zinc is absorbed in the small intestine; absorption is inhibited by the presence of phytates and fiber in the diet, as well as dietary iron and calcium [11].

Anemia in children leads to increased morbidity and mortality [12]. Adverse health effects of anemia in children include impaired psychomotor development and renal tubular function, poor cognitive performance and mental retardation [13,14].

Therefore, this study was done to determine the association of blood lead level > 10 μg/dl, with the increased risk to anemia compared to levels less than 10 μg/dl, also, to investigate the relationship between anemia and changes in blood iron(Fe), zinc(Zn) and copper(Cu) levels, and measure lead level in drinking water.

## Methods

### Study population

This research was carried out on a total of 60 children from the pediatric clinic in Al-Zhraa Univerisity hospital and a special pediatric clinic in a rural area. They were selected by a systematic random sample. Exclusion criteria of cases were children having chronic hemolytic anemia or those suffering from chronic illness associated with anemia. The control group was selected from those attending the out patients clinic for evaluating physical fitness for different sports. Mothers of children were informed about the aim of the study and their consent was obtained. Data related to age, gender, residence, source of drinking water, degree of father and mother's education and their occupation, also, socioeconomic status data was collected from the mothers. According to the WHO definition of anemia based on hemoglobin level less than 11 g/dl, the studied population was divided into anemic and control groups [15]. The anemic group was further classified into categories of mild (Hb level 10-10.9 g/dl), moderate (Hb level 8-9.9 g/dl) and severe (Hb level < 8 g/dl) anemia. Also, according to serum blood level, the studied population was classified into two groups, <10 μg/dl and ≥10 μg/dl.

### Laboratory investigations

A venous blood sample was taken from each child and divided into three tubes. The first tube (containing EDTA) used for estimation of hematological parameters using Celttac autoanalyzer, these parameters included the red blood cell count (RBC), hemoglobin (Hb), hematocrit (Hct), mean corpuscular volume (MCV), mean corpuscular hemoglobin (MCH), mean corpuscular hemoglobin concentration (MCHC), and red cell distribution width (RDW). The second tube (containing heparin) for estimation of lead, copper and zinc by the atomic absorption spectrophotometer [16]. (Perkin Elmer HGA 460-Germany). The blood lead level (BLL) was determined by the graphite furnace atomic absorption spectrophotometer, where as Cu and Zn concentrations were measured with the flame atomic absorption spectrophotometer. The third tube, Hitachi 911 autoanalyzer was used for serum iron estimation using Roche reagent kits whereas Elecsys 1010 - Japan was used for estimating serum ferritin.

### Environmental assessment

Drinking water Samples were taken from tap and hand pump water for detection of lead level using the atomic absorption spectrophotometer (Graphite Furnace, Perkin Elmer HGA-600, USA). Lead level in piped water was found to be 2.9 μg/dl and 3.6 μg/dl in the urban and rural areas, respectively. Hand pump, water collected from two separate hand pumps in the rural area, revealed lead levels to be 3.1 μg/dl and 2.3 μg/dl.

### Statistical analysis

Data was analyzed by SPSS (Statistical Package for Social Sciences) version 12. Chi-square test was performed to compare individual characteristics and the t-test was performed to compare the hematological parameters between anemic and control groups. Results were expressed as the mean ± standard deviation (SD). Significant values of P at < 0.05 and < 0.001 were considered. A correlation was performed for the levels of lead, Fe, Cu and Zn in blood versus the different hematological parameters.

## Results

This study was done on 60 children with ages ranging from 2 to14 years with a mean value of 6.27 ± 3.40 years. According to the blood lead level, ranging between 7 to 20 μg/dl, approximately 63.33% (n = 38) of children had a blood lead ≥10 μg/dl (high blood lead level group {HBLL}) and 36.67% (n = 22) had a blood lead level <10 μg/dl (low blood lead level group {LBLL}), The socioeconomic characteristics were studied among the high and low blood lead level (Table 1). Although, higher BLL was among children >6 years old and those consuming tap water, yet no statistical difference was detected between those having high blood levels. Also, blood lead level was higher in males than females and those of low social standard. In addition, it was higher in children of illiterate mothers and fathers, unemployed mothers and employed fathers.

**Table 1:** Distribution of individual characteristics in relation to blood lead levels.

Characters of studied group	Blood lead level		
	Low <10 μg/dl (n = 22)	High ≥10 μg/dl (n = 38)	Test of significance
	No (%)	No (%)	
**Age**:			
- school children (≥6 years old)	9 (40.9)	20 (52.6)	x^2 ^= 0.76
Pre-school children (<6 years old)	13 (59.1)	18 (47.4)	P = 0.4

**Gender**:			
- Male	9 (40.9)	23 (60.5)	x^2 ^= 2.15
- Female	13 (59.1)	15 (39.5)	P = 0.1

**Residency**:			
- Urban	12 (54.5)	20 (52.6)	x^2 ^= 0.21
- Rural	10 (45.5)	18 (47.4)	P = 1.0

**Mother education**:			
- Education	3 (13.6)	4 (10.5)	x^2 ^= 0.15
- Illiterate	19 (86.4)	34 (89.5)	P = 0.9

**Father education**:			
- Education	3 (13.6)	4 (10.5)	x^2 ^= 0.34
- Illiterate	19 (86.4)	34 (89.5)	P = 0.8

**Mother work**:			
- Employed	3 (13.6)	4 (10.5)	x^2 ^= 0.13
- Unemployed	19 (86.4)	34 (89.5)	P = 0.7

**Father work**:			
- Employed	16 (72.7)	28 (73.7)	x^2 ^= 0.07
- Unemployed	6 (27.3)	10 (26.3)	P = 0.9

**Sources of drinking water**:			
- Tap water	12 (54.5)	20 (52.6)	x^2 ^= 0.02
- Hand pump water	10 (45.5)	18 (47.4)	P = 1.0

**Socioeconomic level**:			
- Middle	3 (13.6)	4 (10.5)	x^2 ^= 0.13
- Low	19 (86.4)	34 (89.5)	P = 0.7

x^2 ^= chi-square test

A significantly greater proportion of children with lead levels ≥10 μg/dl (63.2%) had anemia compared to those with lead levels <10 μg/dl (27.3%) (Table 2).

**Table 2:** Prevalence of anemia in relation to blood lead levels.

	Low blood lead level <10 μg/dl (n = 22) No (%)	High blood lead level ≥10 μg/dl (n = 38) No (%)	Test of significance
No anemia	16 (72.7)	14 (36.8)	x^2 ^= 7.17 p = 0.00
	
Anemia	6 (27.3)	24 (63.2)**	

** p = 0.00

The presence of different categories of anemia among the high (≥10 μg/dl) and low (>10 μg/dl) blood lead level groups was demonstrated in Table 3. The difference in distribution was significant for the mild and severe form of anemia (HBLL 28.9%; LBLL 4.5%) and (HBLL 21.1%; LBLL 4.5%), respectively.

**Table 3:** Distribution of hemoglobin level in relation to blood lead levels.

Categories of anemia according to hemoglobin level	Low blood lead level <10 μg/dl (n = 22) No (%)	High blood lead level ≥10 μg/dl (n = 38) No (%)	Test of significance
No anemia (Hb level ≥11 g/dl)	16 (72.7)	14 (36.8)	x^2 ^= 10.5 p = 0.01
	
Mild anemia (Hb level 10-10.9 g/dl)	1 (4.5)	11 (28.9)*	
	
Moderate anemia (Hb level 8-9.9 g/dl)	4 (18.3)	5 (13.2)	
	
Severe anemia: (Hb level < 8 g/dl)	1 (4.5)	8 (21.1)*	

* p = < 0.05

Comparison between mean values of different hematological parameters and serum ferritin in anemic and control groups were studied (Table 4). Regarding the hematological parameters, nearly all values were significantly lower among the anemic than the control group except for the RDW, which showed a highly significant elevation among the anemic group. As for the RBC count, no statistically significant difference was detected between the groups.

**Table 4:** Comparison between mean values of different hematological parameters and serum level of ferritin in anemic and control groups.

	Anemic group (n = 30) Mean ± SD	Non-anemic control (n = 30) Mean ± SD	Test of significance
RBC (×10^6 ^mm^3^)	4.05 ± 0.51	4.24 ± 0.28	t = -1.7 p = 0.08

Hb (g/dl)	8.97 ± 0.88	12.13 ± 0.46**	t = -17.2 p = 0.00

Hct	28.59 ± 2.26	36.56 ± 2.01**	t = -14.5 p = 0.00

MCV(μ^3^)	70.02 ± 14.80	81.48 ± 6.70**	t = -3.8 p = 0.00

MCH	21.74 ± 3.21	27.08 ± 1.23**	t = -8.5 p = 0.00

MCHC	31.38 ± 1.98	33.54 ± 1.91**	t = -4.2 p = 0.00

RDW	17.27 ± 2.66	13.24 ± 0.60**	t = 8.0 p = 0.00

Ferritin (ng/ml)	40.21 ± 23.46	82.28 ± 9.76**	t = -9.0 p = 0.00

** p = 0.00

t = t-test

**Figure 1:**
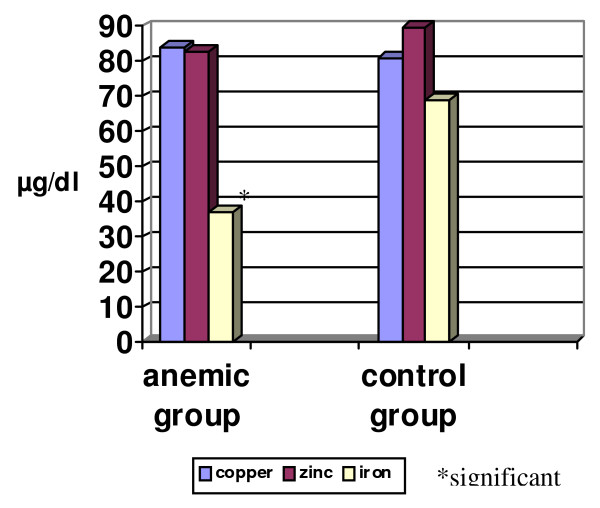
**Comparison between mean values of copper, zinc and iron levels in anemic and control groups**.

**Figure 2:**
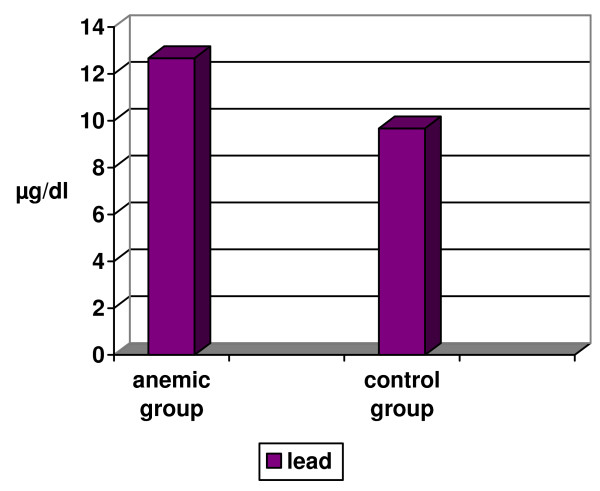
**Comparison between mean values of blood lead levels in anemic and control groups**.

Comparing the results of Cu, Fe and Zn levels between the anemic and control groups revealed a significant decrease in the level of Fe among the anemic than the control group (p < 0.001). Whereas no statistically significant difference was seen between both groups for Cu and Zn levels (Fig 1). As for the BLL between anemic and control groups, a significantly high BLL was found among anemic in comparison to control group (p < 0.001; Fig 2).

Table 5 reveals the correlation between the different hematological parameters and the blood levels of lead, Cu and Zn. According to lead a significant negative correlation is seen between it and Hb, Hct, MCV, MCH, Fe, and ferritin (r = -0.461, r = -0.484, r-0.267, r = -0.381, r = -0.470 and r = -0.552, respectively) (p < 0.001). Whereas a significant positive correlation existed between it and RDW (r = 0.458; p = 0.001). In addition, Cu showed a positive significant correlation with RBC (r = 0.264; p < 0.05) and a negative significant correlation with ferritin (r = -0.257; p < 0.05). More over Zn levels revealed a positive significant correlation in relation to Hb, Hct, MCH and ferritin (r = 0.324, r = 0.305, r = 0.308 and r = 0.314) and a negative significant correlation with RDW (r = -0.266) (p < 0.05).

**Table 5:** Correlation of different hematological parameters, serum iron and ferritin levels in relation to blood lead, copper and zinc.

Hematological parameters	Blood lead level r-value (p-value)	Copper r-value (p-value)	Zinc r value (p-value)
RBC (×10^6 ^mm^3^)	0.118 (0.37)	0.264* (0.04)	0.015 (0.90)

Hb (g/dl)	-0.461** (0.00)	-0.159 (0.22)	0.324* (0.01)

Hct	-0.484** (0.00)	-0.209 (0.11)	0.305* (0.01)

MCV(μ^3^)	-0.267* (0.03)	-0.177 (0.17)	0.248 (0.05)

MCH	-0.381** (0.00)	-0.156 (0.23)	0.308* (0.01)

MCHC	-0.155 (0.23)	-0.020 (0.87)	0.120 (0.36)

RDW	0.458** (0.00)	0.238 (0.06)	-0.266* (0.04)

Ferritin (ng/ml)	-0.552** (0.00)	-0.257* (0.04)	0.314* (0.01)

Fe (μg/dl)	-0.470** (0.00)	-0.136 (0.30)	0.186 (0.15)

* p = < 0.05

** p = 0.00

r = correlation coefficient

Assessment of mean values of iron (Fe) and ferritin among the low and high blood lead level groups revealed (Table 6) significantly lower blood iron and ferritin levels in the high BLL group than those of the low BLL group (48.86 ± 19.05 and 51.32 ± 24.52, 60.80 ± 21.27 and 78.39 ± 24.70, respectively).

**Table 6:** Mean values of serum iron (Fe) and ferritin in relation to blood lead levels.

	Low blood lead level <10 μg/dl (n = 22) Mean ± SD	High blood lead level ≥10 μg/dl (n = 38) Mean ± SD	Test of significance
Fe (μg/dl)	60.80 ± 21.27	48.86 ± 19.05*	t = 2.24 p = 0.02

Ferritin (ng/ml)	78.39 ± 24.70	51.32 ± 24.52**	t = 4.10 p = 0.00

* p = < 0.05

** p = 0.00

## Discussion

More than half of the study children (63.33%) had BLL ≥10 μg/dl, similar to a study done by Jain et al [6]. who also reported a significant association of moderate and severe anemia with 10-19.9 μg/dl blood lead levels. while the present study reported a significant association of mild and severe anemia with 10-20 μg/dl blood lead levels, the difference in results may be due to a small sample size in the present study. However the current study is similar to the estimation obtained for children in India [17,18]. The cutoff value of l0 μg/dl defined by the Center for Disease Control and Prevention as a limit for an elevated blood lead level primarily is based on neurological toxicity [19]. Recently, no level less than 10 μg/dl is considered safe [4].

Schwartz et al [20]. reported that children living near primary lead smelters in the US of Idaho, had blood lead levels near 25 μg/dl and were associated with anemia in a dose-related manner. In addition, Drossos et al [21]. reported that children with BLL >30 μg/dl had a linear decline in hemoglobin level. Whereas on the contrary, Froom et al [22]. suggested that hemoglobin level did not correlate well with BLL and suggested that anemia is not related to lead at low BLL. However, other studies reported a variable association [23-27].

Lead causes anemia by impairing heme synthesis and increasing the rate of red blood cell destruction [28]. On the other hand, it is also possible that iron deficiency, which is a proven cause of anemia, leads to increase in the absorption of lead in the body, resulting in high BLL [29,30]. Although a causal pathway cannot be determined, yet the study findings clearly demonstrate an association between varying severity of anemia and elevated BLL.

In the current study high BLL among school children (>6 years old) may be due to usage of crayon in school and high BLL in male children than female children may explain by more hobbies in males.

Fe, Cu and Zn are essential elements for the maintenance of life and health. Pb which is a heavy metal, can be harmful to human health. Therefore, the blood level of these elements in children was determined. Because of the presence of high BLL in drinking water, as reported by the WHO, this study was carried out to reveal the relationship between high blood lead levels, trace elements as well as hematological parameters in children.

In the present study, the level of iron in the anemic group was found to be significantly lower than the control as was expected, similarly Jain et al [6]. represented measure finding. As Fe has an essential role in many biological processes and as deficiency is a World health problem, especially for infants and rapidly growing adolescents. Therefore, it is important to maintain iron concentration within its narrow normal range [31].

In the present study serum Zn level of the anemic group is insignificantly lower than the control group. There is an antagonism between Zn and Fe absorption from the gastrointestinal tract, as an increase iron concentration in the intestinal lumen may antagonize the uptake of Zn [32]. A study done by Sebahat et al [1]. found a decrease in serum Zn level and an increase in serum Cu level in the anemic group compared to the control group.

In accordance, although the present study revealed the Cu level to be higher in the anemic more than control group yet, this increase was not statistically significant. However, Cu has a role in the absorption of iron. The oxidation of ferrous iron into ferric state is carried by ceruloplasmin. This depletion of Cu could impair iron absorption [33].

In the present study, the serum level of Pb in the anemic group was significantly higher than in the control. A possible explanation is that Fe deficiency increases absorption of Pb from the intestines. Similarly a study carried out in Canada, revealed high BLL in babies with Fe deficiency [25]. Other studies revealed significant associations between Fe deficiency and high blood lead level [30,34].

The current results showed that Hb, Hct, MCV, MCH and ferritin values of children with anemia decreased and RDW level increased in comparison to control group.

Also, blood lead levels were higher in anemic children. This could be due to that decreasing iron level increases lead absorption that in turn affects heme synthesis, thus negatively affecting hematological parameters [28]. Moreover high BLLs were found to be associated with lower iron and ferritin levels than lower lead levels. This may be that iron absorption occurs predominantly in the duodenum and jejunum. Also, a number of dietary factors influence iron absorption, where ascorbate and citrate increase its uptake. Lead in particular is a pernicious element to iron metabolism. As it is taken up by the iron absorption machinery instead of iron, and through competitive inhibition. Further more, it interferes with a number of important iron dependent metabolic steps such as heme biosynthesis [2].

In investigated water samples were considered suitable for drinking according to the EMH [35]. as the lead level was lower than the permissible limit (5 μg/dl), although the WHO [36]. considered higher than the permissible limit to be 1 μg/dl. In Dakahlya-Egypt, lead level in drinking water was higher than the permissible limit according to the WHO. In Egypt, the control of lead is not efficient, so that the level of lead in drinking water in some sporadic areas is still high level [37].

In 2003-2004, tap water in Washington, DC, exceeded the Environmental Protection Agency (EPA) regulations. This was because of a change in water disinfection procedures, which increased the water ability to leach lead from connector pipes between water mains and interior plumbing in old houses [38].

In developing countries such as India, control of lead pollution is much slower and more sporadic. Some studies estimated that more than half of children in India have blood lead levels > 10 μg/dl [18].

The present work revealed an association between blood lead level and low serum iron and ferritin levels. This is similar to several studies reporting higher proportions of children with elevated blood lead levels among those with low iron and ferritin levels [39-41]. These results suggest that inadequate iron status may amplify the effect of lead contamination in the environment by increasing absorption and possibly retention of lead in the body [39].

On the contrary Hershko et al [42], reported a lack of correlation between iron and blood lead in older children.

## Conclusion

In the present study, lead levels ≥10 μg/dl in children were associated with an increased risk of mild and severe anemia, decreasing iron absorption and negatively affecting the hematological parameters. High BLLs were associated with low blood level of iron and ferritin. Lead level in drinking water was high according to the WHO, and this may be one of the leading causes for elevating BLL in children. Lead pollution might be controlled and steps should be taken to reduce the prevalence of childhood anemia.

## Competing interests

The authors declare that they have no competing interests.

## Authors' contributions

AAH contributed to the study design, acquisition of data, analysis and interpretation of data, and drafted the manuscript. MMZ contributed to the study design, acquisition of data. MAA contributed to study design and interpretation of data, and drafted the manuscript. AAM contributed to the revision of the manuscript. RAS contributed to study design. All authors read and approved the final manuscript.
